# In vitro measurement of temperature changes during implantation of cemented glenoid components

**DOI:** 10.3109/17453671003717823

**Published:** 2010-04-06

**Authors:** Patric Raiss, Guido Pape, Sebastian Jäger, Markus Loew, Rudi Bitsch, Markus Rickert

**Affiliations:** Department of Orthopaedics, University of HeidelbergGermany

## Abstract

**Background and purpose** It is unclear whether the increase in temperature during cement curing may cause osteonecrosis, leading to loosening of the glenoid component in shoulder arthroplasty. We therefore analyzed the temperature during implantation of cemented glenoid implants.

**Methods** 8 keeled and 8 pegged glenoids were implanted in standardized fashion in 8 pairs of scapulas. Temperature and pressure sensors were implanted at the bone-cement interface in the glenoid. Real-time measurements were made of temperature and pressure within the glenoid vault.

**Results** In no case was the temperature reached high enough to endanger the surrounding bone. The mean increase in temperature was 5° (0.5–6.9) in the keeled group and 2.7° (1.7–3.6) in the pegged group. The mean maximum pressure in the keeled group was 50 kPa (20–100) and in the pegged group it was 113 kPa (60–181). Both differences were statistically significant.

**Interpretation** The temperatures that occur during implantation of cemented components are low and probably not high enough to cause osteonecrosis in the surrounding bone.

## Introduction

Loosening of the glenoid component is a major complication in total shoulder arthroplasty (Hayes and Flatow 2001, Burroughs et al. 2003, Sperling et al. 2004, Bohsali et al. 2006, Chin et al. 2006). Multiple factors such as eccentric loading (Franklin et al. 1988), poor bone quality (Frich et al. 1997), poor cementing technique (Norris and Lachiewicz 1996), and high activity levels (Nyffeler et al. 2006) have been described as reasons for loosening.

Multiple implant concepts such as cemented or uncemented glenoid components and different implant designs with pegs, keels, or screws have been developed in recent years. High temperatures were measured at the implant surface in cemented glenoid components during implantation by Churchill et al. (2004), who thought that this could put living bone at risk and that thermal osteonecrosis may be a cause of progressive radiolucent lines. Radiolucent lines have been observed on radiographs in up to 95% of patients at follow-up (Wirth and Rockwood 1996, Torchia et al. 1997, Lazarus et al. 2002).

Cement penetration into bone depends, among other considerations, on the pressure during implantation of the component (Sih et al. 1980). A large volume of cement has been suggested to endanger bone by leading to thermal osteonecrosis (Huiskes 1980).

Our first hypothesis in this in vitro study was that temperatures during implantation of cemented glenoid components may be high enough to endanger the surrounding bone. The second hypothesis was that different implant designs may have an influence on the temperatures and pressures.

## Material and methods

### In vivo measurements of temperature in glenoids

The behavior and the increase in temperature of bone cement depend on the local temperature where the cement is used. Thus, in vivo measurements were performed to determine the temperature of glenoids during shoulder replacement surgery. In 10 shoulders the temperature of the glenoid surface was measured 10 times, in each case using the infrared thermography Inspacto 900 plus (Infrapoint, Saalfeld, Germany) after preparation of the bone before and after the use of Jet-Lavage (Biomet, Warsaw, IN). After using the Jet-Lavage there was no bleeding of the bone bed.

The mean temperature of the glenoid surface before using the Jet-Lavage was 29.4° (27.2–31), and thereafter it was 26.2° (25–27.5). These data were used in the present study.

The study was approved by the ethics committee of our local university (number: S-304/2007).

### Origin, preparation, and implantation of glenoid components

**Figure 1.**
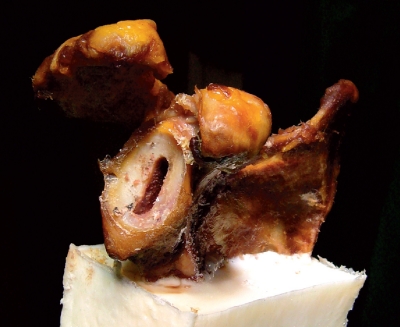
Workstation with a prepared scapula fixed in resin in a screw clamp.

We used 8 pairs of fresh frozen scapulas obtained from the International Institute for Advancement of Medicine (Jessup, PA). Each pair comprised 2 scapulas from 1 donor. All soft tissues were dissected from the bone. For preparation of the glenoid surface, the scapulas were fixed in a 2-component synthetic resin (RenCast FC 53 A/B; Goessl and Pfaff, Karlskron, Germany). A special 7 × 12.5 × 15 cm metal form was constructed to fix the scapulas in a standardized fashion. After curing of the synthetic resin, the preparation was placed in a screw clamp to ensure adequate fixation for the preparation of the glenoid (Figure 1). Afterwards, the frozen scapulas were warmed in a heat box for 12 h.

**Figure 2.**
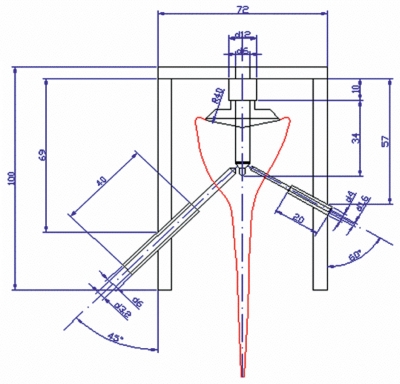
Engineering drawing of the drill jig.

In no case were severe glenoid erosions found. In all cases, both the preparation and the implantation of the glenoids were performed by the same experienced surgeon. After locating the glenoid center and drilling the center hole, the glenoid surface was reamed with spherical reamers of increasing size as recommended by the manufacture (Tornier, Edina, MN). A special drill and fixation jig (Figure 2) for preparation of the holes for the pressure and temperature sensors was placed into the center hole. The drill jig has a center peg for fixation in the center hole and a positioning frame with drill sockets on both sides. Considering the anatomy of the scapula, the hole for the temperature sensor was made using a 3.2-mm drill at a 30° angle on the spinal side of the scapula. The hole for the pressure sensor was made using a 1.6-mm drill at a 45° angle on the coracoid side. The holes for the sensors were drilled as far as the central drill hole. Afterwards, preparation of the glenoids was continued.

In one scapula from each donor a pegged glenoid and in the other scapula a keeled glenoid was implanted in randomized fashion (Tornier, Edina, MN, USA). A testing component was inserted in all cases, as recommended by the manufacturer. To remove any loose debris in the glenoid keel or peg holes, 200 mL of Jet-Lavage was used in each case.

**Figure 3.**
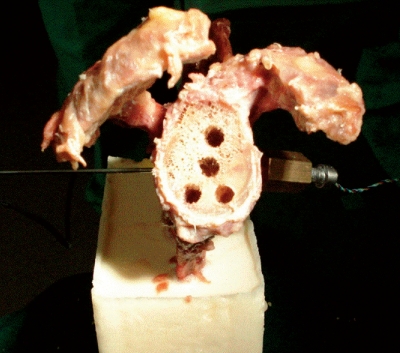
Frontal view of a prepared glenoid with the pressure sensor on the coracoid side and the temperature sensor on the spina side.

Next, the sensors were put in place (Figure 3). The miniature pressure sensor (XPR36; Disynet, Brueggen, Germany) was inserted into a cylindrical holder and fixed in the scapula. This sensor device was hermetically filled with wax as a transmitter (Goessl and Pfaff) to avoid direct contact between the sensor surface and the cement. The sensor has a measurement range of 0–200 kPa.

The temperature sensor (Pt 100; B+B Thermo-technique, Donaueschingen, Germany) was fixed in the hole on the spinal side of the scapula. This sensor has a measuring accuracy of 0.1°C between –50°C and +600°C. Pressure and temperature data were recorded in real time by means of customized data-logging software. Taking the data from the literature into account (Nyffeler et al. 2006), we did not distinguish between anterior or posterior placement of the sensors, although we were aware that the anterior scapula wall is thinner than the posterior one.

The tips of both sensors were placed at the inner surface of the previously prepared glenoid vault. This represents the top of the center peg (pegged components) and the posterior surface of the keel (keeled components). Positioning was performed under visual control so that the sensors could be placed exactly at the bone-cement interface.

**Figure 4.**
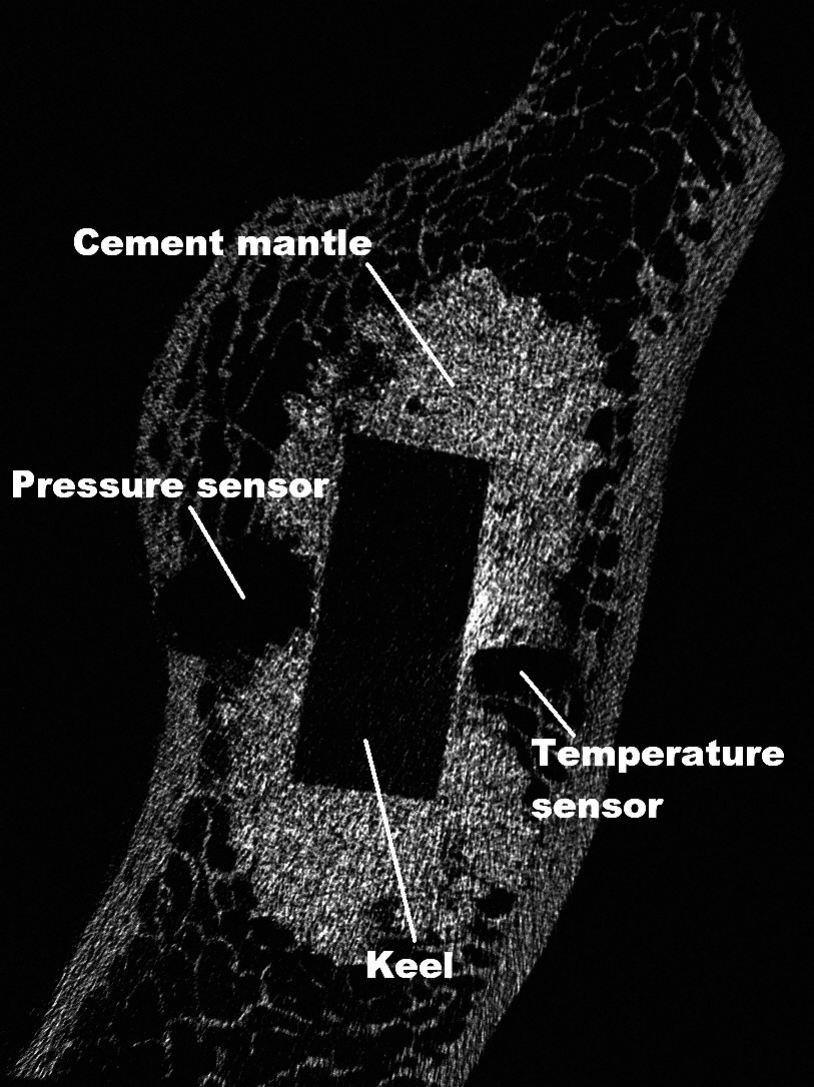
Frontal micro-CT scan of a glenoid cavity showing a continuous cement mantle around the keel. The drill holes where the pressure and temperature sensors were placed are showing that they had direct contact with the cement.

Post-experimental micro-CTs (with a resolution of 35 μm) showed that in all cases the sensors for temperature and pressure had been placed at the bone-cement interface with direct contact to the cement (Figure 4).

### In vitro measurement of pressure and temperature

Low-viscosity, 2-component bone cement (Palacos; Heraeus, Wehrheim, Germany) was used. The measurements were started with cement mixing at a bone temperature of 26.2°C, as measured with the temperature sensor. We used a vacuum-mixing system, including a vacuum pump, a manometer, an air-pressure system, and a cement gun (Heraeus). The mixing time for the bone cement was 30 s as recommended by the manufacturer, at a constant room temperature of 22°C. After mixing of the components, the cement was filled into a 5-mL syringe and weighed. After 4 min, the cement was placed into the prepared drill holes for pegged components or into the keel slot for keeled components. Then, 1 mL of cement was placed on the posterior surface of the components as recommended by Nyffeler et al. (2006). The glenoid components were pressed manually into the prepared scapulas after 5 min. Constant pressure was applied by the same surgeon, with the use of an impactor in all cases. To measure the force during impacting of the components, the impactor was modified and included a force sensor (FN 3060; Disynet). The mean force during impaction was 145 N (maximum 188).

The excess cement was collected and added to the remaining cement in the syringe. Its weight was subtracted from the weight measured initially to calculate the amount of cement used. The measurements with the constant pressurization ended after 20 min.

### Statistics

Statistical analysis was performed using SPSS 15.0. The Wilcoxon test was used to assess differences in increase of temperature and pressure during implantation of the glenoid components. Differences were considered significant at p < 0.05.

## Results

In no case did we measure temperatures of 47°C or more. Significant differences were found for the mean increase in temperature (peg vs. keel) and between the mean maximum pressure (peg vs. keel) during implantation of the components (p < 0.05 in both cases).

The mean increase in temperature measured in the pegged group was 2.7° (1.7–3.6) and it was 5° (0.5–6.9) in the keeled group. The difference between the groups was significant (p < 0.03).

The mean maximum pressure during implantation was 113 (60–181) kPa in the pegged group and 50 (20–100) kPa in the keeled group (p < 0.05) (Table). Fractures of the trabecular bone were not found on the micro-CT scans.

**Table:** Measurements of the maximum pressures occurring during implantation of the keeled and the pegged components

Group measured	Specimen no.								
	1	2	3	4	5	6	7	8	Mean
Keel (kPa)	43	37	42	97	37	20	87	39	50
Peg (kPa)	60	69	105	114	165	181	76	131	113

Because the keel implant itself has a lower volume than the 4 pegs, more cement was needed for a complete filling in the peg group. The mean weight of cement used for filling the peg holes was 2.26 (1.65–2.5) g. A mean of 1.13 (0.98–1.29) g of cement was applied to the posterior surface of the component. The mean weight of cement used for filling the keeled slot was 2.42 (1.86–2.61) g. A mean of 1.1 (1.01–1.23) g of cement was applied to the posterior surface of the component.

## Discussion

The temperatures that occur during cement curing have been described as a possible cause of thermal necrosis of the bone (Churchill et al. 2004, Stanczyk and van Rietbergen 2004, Hsieh et al. 2008). Eriksson and Albrektsson (1983) stated that a temperature of 47°C for 5 min, 50°C for 1 min, or 56°C for less than 1 min results in immediate bone necrosis. Thus, we defined a temperature of 47°C or more as being potentially capable of putting bone at risk. Temperatures of up to 99°C in patients with bone cysts have been measured during cement curing in hip arthroplasty (Hsieh et al. 2008).

Sih et al. (1980) showed that there is a linear relationship between the increase in temperature during the exothermic reaction of cement curing and the amount of cement used, and they stated that a cement mantle thickness of more than 5 mm would be a risk factor for thermal bone necrosis in hip arthroplasty. A positive correlation between pressure and cement penetration has been shown in hip arthroplasty (Oates et al. 1995, Parsch et al. 2004). Because of these relationships between pressure and temperature when using bone cement, we measured both parameters.

In a finite-element model of the femur, Jansson et al. (1993) showed that the thickness of the cement mantle is influenced by pressure in hip arthroplasty. A pressure of 100 kPa was recommended for sufficient and ideal cement penetration into the bone. In an experimental study, Bitsch et al. (2008) found that pressures of around 30 kPa during implantation of hip resurfacing implants led to an adequate cement mantle.

The mean pressures we found were 113 kPa for the pegged group and 50 kPa for the keeled group. To our knowledge, there have been no studies published regarding the pressures occurring during implantation of cemented glenoid components. Thus, our results can only be compared to the findings with other joints, as mentioned above. The relatively high pressures are explained by the physical relationship between pressure, force, and area. Pressure is defined as force per unit area. Because the surface area of the central peg is smaller than that of the keel, the quotient of force per unit area is greater for the peg group. Compared to other studies dealing with pressures in cemented arthroplasties, the pressures occurring in the present investigation appear to have been sufficient to ensure adequate penetration of the cement into the surrounding bone.

We have found only one study on temperatures during the implantation of cemented glenoid components (Churchill et al. 2004). The findings of that study differ from ours, because that group performed the glenoid implantations at higher bone temperatures (37°C, as opposed to 26°C in our study). We do not believe that the higher amounts of bone cement in Churchill's study (4.88 g vs. 3.39 g in pegged components and 5.87 g vs. 3.52 g in keeled components) are responsible for the divergent results.

The starting temperature of the bone has an important influence on the increase in temperature of the cement, which may be one important reason for the different findings. We found in our experiment that the in vivo temperature of the glenoid bone after preparation and use of a jet lavage is substantially lower (26°C) than the physiological body temperature chosen by Churchill et al. (2004); the former results in a lower increase in temperature during cement curing.

Our study has several limitations. First, we were able to measure temperatures at one point of the bone-cement interface only and not at several positions. This limitation is related to the small size of the glenoid bone and the size of the temperature sensors, which makes it technically impossible to place more than one or two sensors. Moreover, the number of specimens tested was low. However, there have been no similar studies with higher numbers published in the recent literature. Other cements and other cementing techniques were not examined. Due to the nature of this experimental study, we have no information about the possible changes of temperature and pressure in vivo. For example, the blood flow within the bone may influence the temperatures reached during cement curing.
